# Intracranial Angioplasty and Stenting before and after SAMMPRIS: “From Simple to Complex Strategy – The Chinese Experience”

**DOI:** 10.3389/fneur.2014.00129

**Published:** 2014-07-14

**Authors:** Zhongrong Miao

**Affiliations:** ^1^Beijing Tiantan Stroke Center, Beijing Tiantan Hospital, Capital Medical University, Beijing, China

**Keywords:** ischemic stroke, intracranial atherosclerotic disease, angioplasty, balloon, stenting, medical therapy

## Abstract

Intracranial atherosclerotic disease (ICAD) accounts for 33–50% of all ischemic strokes in the Asian population (1) and represents an important public health issue in China. The results of the SAMMPRIS trial alarmed most experienced interventionalists in China for two reasons. Firstly, the high complication rate in the stenting arm (20% the first year) was higher than expected. Secondly, the recurrent stroke rate in the aggressive medical treatment arm at 12.2% during the first year was unacceptably high, not to mention the fact that such tight vascular risk factor control is difficult to achieve for many patients in real life clinical experience, at least in China. The experience of treating ICAD in China, gained over the last two decades, is very rich and promising. We intend to highlight these past experiences and address future trials and trends in China. We will also address our criticism of the SAMMPRIS trial design in order to better design a future trial.

## Intracranial Stenting for ICAD in China before SAMMPRIS

The warfarin–aspirin symptomatic intracranial disease (WASID) study showed that the role of medical therapy for intracranial atherosclerosis (≥70%) is less effective, with the 1-year risk of ischemic stroke remaining as high as 23% in patients who presented with stroke and 14% in patients who presented with transient ischemic attack (TIA) (2). Inspired by experience from the treatment of the coronary artery disease, Chinese doctors began treating patients with symptomatic intracranial artery stenosis refractory to medical therapy with endovascular treatment since the 1990s. The devices initially used were the coronary balloons Magellan (Balt Co., Montmorency, France) and SeQuent (B. Braun, Melsungen, Germany). Different stents including Coroflex or Coroflex Blue (B. Braun, Melsungen, Germany), BiodivYsio (Biocompatibles Ltd., Farnham, UK), S660 (AVE, Galway, Ireland), and Firebird (MicroPort, Shanghai, China) were also used. Initial reports were all single-center, self-reported studies, with varying degree of success (96.46–97.6%) and low complication rates (4.42–10%). When examined together, these studies included a total of 528 patients (3–9), which constituted a rich database. When compared with the expected natural history of the disease, the consensus at that time was that these results supported the use of the coronary stents as a mean for stroke prevention in patients with intracranial atherosclerotic disease (ICAD).

With the dramatic increase of new cases, new devices specifically designed for ICAD were developed. The Wingspan stent system (Stryker, Kalamazoo, MI, USA) was the first commercially available device since 2005 (10) and was introduced to China after approval by the State Food and Drug Administration (SFDA) in 2007 (11). The Apollo balloon-mounted stent (MicroPort, Shanghai, China) was also approved by the SFDA the following year. Since then, a series of registries followed their introduction to the market, showing promising results for both the Wingspan and Apollo stent systems (11, 12).

The current Chinese experience was summarized best in the last Tiantan International Stroke Conference (TISC). A poll analysis was presented on 1372 treated lesions between March 2005 and November 2011 using different devices (13). The distribution of these lesions was as follows: 91 (7%) at the distal internal carotid artery (ICA), 795 (58%) at the M1 segment of the middle cerebral artery (MCA), 239 (17%) at the basilar artery (BA), and 247 (18%) at the intracranial vertebral artery (VA). Devices used included 323 coronary stents, 109 specially made intracranial balloon-mounted stents (Apollo), 638 Wingspan stents, and 38 cases of balloon angioplasties alone. The success rate was promising with an average rate of 96% (92–100%). The complication rate within 30 days was 8% (from 3.2 to 14.8%) (13).

We also independently reported our prospective registry focused on symptomatic MCA stenosis, which demonstrated a relatively high 1-year complication rate of 19.4%, compared to the medical group of 17.6% (*p* = 0.85) (14). This result was very similar to the later published SAMMPRIS study. Then, the general consensus was that stenting is feasible, but its effectiveness at preventing recurrent stroke with high grade symptomatic intracranial stenosis still needed validation (7, 9, 10, 12, 13, 15).

## Since SAMMPRIS

The publication of the SAMMPRIS (16) results, the first and only prospective randomized trial to date, demonstrated high complication rates in the first 30 days following the Wingspan stenting in one arm and lower than expected stroke risk in the aggressive medical treatment (AMT) arm. This changed the accepted belief of the efficacy of intracranial stenting as a measure of stroke prevention, and there is no reason to believe that a similar prospective trial using the same device will have different results in China. Based on the results, it is estimated that for intracranial stenting to remain a promising measure for stroke prevention in these patients, the peri-procedural complication rate within the first 30 days needs to be <4% (7, 13). From our personal experience, and reviewing the above highlighted Chinese experience, we believe that in order for us to obtain such a low complication rate we need to enact a few important changes. Firstly, a different patient selection criterion should be employed. Secondly, a more complex treatment strategy needs to be adopted (not all vessels or lesions are the same). Finally, we need a better device than the Wingspan stent system (7).

The most obvious conclusion of SAMMPRIS is that the device exclusively used (the Wingspan system) is not well suited for intracranial stenting. We can speculate as to why the Wingspan system ended up not being suitable for intracranial stenting, such as the need for two steps (angioplasty then stenting) or the low radial force of the stent making its opposition to the arterial wall very limited, which has a tendency to encourage platelet aggregation and clot formation beneath the stent (17, 18). Besides the stent itself, we believe that there was another shortcoming in the trial design (19). Dissection following angioplasty has been shown in a prospective registry to predict a higher stroke rate in the peri-procedural period (20, 21). Since the slow inflation technique of the angioplasty balloon was not included in the trial protocol, and no angiogram following the angioplasty was obtained prior to the stent placement, we can speculate that some of the complications in SAMMPRIS were due to unaccounted dissections caused by suboptimal angioplasty technique (7, 13, 17, 19). Secondly, in SAMMPRIS all the vessels were grouped together without distinction between vessels with perforators (BA, MCA) and those without perforators (VA, ICA), despite their known different complication rates (20, 21). Thirdly, the SAMMPRIS protocol did not take into account the Mori classification, yet there are numerous papers showing that lesions with different characteristics as classified by Mori carry different risks during intracranial endovascular revascularization (IER) (20–26). However, we still believe that SAMMPRIS was an important study because at least it forced us to examine the question: how safe is IER?

Building on prior literature, future trials need to take into account the following points:

1. *Improve patient selection*: which group of patients will most likely benefit from IER? It is suggested that patients with poor collaterals stand a higher chance of benefit from IER than patients with excellent collaterals (22). Poor collateral circulation is determined as ≥40% decrease in cerebral blood flow (CBF) at the stenotic arterial territory compared to CBF at the reference area by CT or MRI perfusion (reference area being defined as the contralateral hemisphere for anterior circulation lesions or anterior circulation territory for posterior circulation lesions); or a ASITN/SIR collateral flow grading system score <3 as confirmed by diagnostic cerebral angiogram (2–26).
2. *Improve device selection:* we believe that different lesions respond better to different devices.
  - For Mori A lesions with straightforward arterial access, the balloon-mounted stent is our first choice, since no exchange maneuver is needed and requires shorter procedural time (3, 27–29).
  - For Mori B or C lesions with tortuous arterial access, or lesions with a significant mismatch in the diameter between the proximal and distal segment, the gateway balloon plus Wingspan stent system is preferred because it is more flexible compared to the balloon-mounted stent system (6–12, 28, 29).
  - For lesions near the perforator vessels (the mid-basilar artery and distal M1 segment), lesions with tortuous arterial access and Mori A classification, or lesions in a target vessel with small diameter (<2.5 mm), angioplasty alone is simpler and safer than stent implantation (28).

## Studies in China Since SAMMPRIS

We recently published our new, prospective, single-center study applying the aforementioned criteria (28). Between November 2011 and October 2012, 158 patients were enrolled into the study and were divided into 3 groups: balloon-mounted stents (BS) group (81 patients, some patients were treated first with gateway balloon and then with the Apollo stent), angioplasty alone (AG) group (39 patients), and balloon angioplasty and Wingspan stenting (AS) group (38 patients). The primary endpoints were successful procedural rate and any vascular event within 30 days. Overall technical success rate was 96% (152/158). Intracranial stenting was successful in 97.5% (79/81) of patients in BS group, 100% (38/38) in AS group, and 89.7% (35/39) in AG group with significant differences between the three groups (*p* = 0.042). The 30-day composite stroke or death rate was 4.4% (7/158). Any stroke or death rate within 30 days in the BS group was 4.9%, in AS group was 7.9%, and 0% in angioplasty AG group (see Table 1). In this study, 59% of angioplasty cases needed secondary stenting due to large dissection. These results, especially in the angioplasty arm, are seemly very encouraging and point the merit of our IER strategy; the primary outcome was not satisfying and more than 50% require secondary stenting. There are more works we should do.

**Table 1:** **The efficacy endpoints of different therapy groups**.

	BS group *n* = 81	AG group *n* = 39	AS group *n* = 38	Total *n* = 158	*p*
**Primary endpoints**
Successful PTAS, *n* (%)	79 (97.5)	35 (89.7)	38 (100.0)	152 (96.2)	0.042
**Secondary endpoints**
Any stroke or death	4 (4.9)	0 (0.0)	3 (7.9)	7 (4.4)	0.231
Any ischemic stroke	4 (4.9)	0 (0.0)	2 (5.3)	6 (3.8)	0.359
Any hemorrhagic stroke	0 (0.0)	0 (0.0)	1 (2.4)	1 (0.6)	0.204
Death	0 (0.0)	0 (0.0)	0 (0.0)	0 (0.0)	–
MI	0 (0.0)	0 (0.0)	0 (0.0)	0 (0.0)	–
SAE	0 (0.0)	0 (0.0)	1 (2.4)	0 (0.6)	0.204
mRS ≥ 3	2 (2.5)	0 (0.0)	2 (5.3)	4 (2.5)	0.339

*BS group, balloon-mounted stent group; AG group, angioplasty group; AS group, angioplasty plus self-expanding stent group; MI, myocardial infarction; mRS, modified Rankin scale; PTAS, percutaneous transluminal angioplasty and stenting; SAE, serious adverse even (28)*.

Currently, there are two ongoing multicenter clinical trials supported by both government agencies and medical device companies: Wingspan Stenting for Symptomatic Intracranial Artery Stenosis Registry study in China (WIRE-CHINA) and Apollo Balloon-Mounted Stent for Symptomatic Intracranial Artery Stenosis Registry study in China (AIRE-CHINA) (7, 13). These studies will be carried out in more than 20 centers. The primary objective is to evaluate the safety of intravascular stenting during the 30-day perioperative period in patients with symptomatic intracranial artery stenosis in the Chinese population using a specific device. Their results are eagerly awaited.

## Conclusion

In patients with symptomatic intracranial atherosclerotic lesion, complex treatment strategy is needed. Different patients have different risk factors and indications, while different lesions respond better to different devices. Future trials are needed and we are very optimistic about their final outcome.

## Conflict of Interest Statement

The author declares that the research was conducted in the absence of any commercial or financial relationships that could be construed as a potential conflict of interest.
